# Abdominal obesity and prolonged prone positioning increase risk of developing sclerosing cholangitis in critically ill patients with influenza A-associated ARDS

**DOI:** 10.1186/2047-783X-17-30

**Published:** 2012-12-22

**Authors:** Thomas Weig, Mirjam I Schubert, Norbert Gruener, Michael E Dolch, Lorenz Frey, Jens Miller, Thorsten Johnson, Michael Irlbeck

**Affiliations:** 1 Department of Anaesthesiology, Ludwig-Maximilians-University, Munich, 81366, Germany; 2 Department of Clinical Radiology, Ludwig-Maximilians-University, Munich, 81366, Germany; 3 Department of Medicine II, Ludwig-Maximilians-University, Munich, 81366, Germany

**Keywords:** Intraperitoneal fat, Multi-organ dysfunction syndrome, Obesity, Prone position, Sclerosing cholangitis in critically ill patients

## Abstract

**Background:**

Secondary sclerosing cholangitis is a severe disease of the biliary tract. Over the last decade, several cases of sclerosing cholangitis in critically ill patients (SC-CIP) were reported. Reports in the literature so far are characterized by a wide variety of underlying causes of critical illness, thereby hindering a risk-factor analysis. We report on a homogenous cohort of critically ill patients with influenza A (H1N1) pneumonia and severe acute respiratory distress syndrome (ARDS), of whom a subgroup developed sclerosing cholangitis, allowing for probing of risk factors associated with SC-CIP.

**Methods:**

Twenty-one patients (5 female, 16 male, 46.3 ± 10.8 years) with severe ARDS due to H1N1 pneumonia were retrospectively divided into two groups, characterized by the presence (n = 5) and absence of SC-CIP (n = 16). A large array of clinical data, laboratory parameters, and multi-detector computed tomography-derived measures were compared.

**Results:**

Both patient groups showed severe pulmonary impairment. Severity of disease on admission day and during the first 14 days of treatment showed no difference. The patients developing SC-CIP had a higher body mass index (BMI) (37.4 ± 6.0 kg/m^2^ vs. 29.3 ± 6.8 kg/m^2^; *P* = 0.029) and a higher volume of intraperitoneal fat (8273 ± 3659 cm^3^ vs. 5131 ± 2268 cm^3^; *P* = 0.033) and spent a longer cumulative period in the prone position during the first 14 days (165 ± 117 h vs. 78 ± 61 h; *P* = 0.038).

**Conclusion:**

Our results suggest that obesity, intraperitoneal fat volume, and a longer cumulative duration spent in the prone position may put patients with ARDS at risk of developing SC-CIP. These results lead us to propose that the prone position should be carefully deployed, particularly in abdominally obese patients, and that frequent checks be made for early hepatic dysfunction.

## Background

Secondary sclerosing cholangitis in critically ill patients (SC-CIP) has only recently been described as a complication in patients suffering from severe life-threatening illness requiring prolonged intensive care treatment. In the first review of SC-CIP, it was suggested that mechanical ventilation with high positive end-expiratory pressure (PEEP) and low tidal volume in combination with vasoconstrictor therapy might promote arterial hypoperfusion of the peri-biliary vascular plexus, causing ischemic injury to bile duct epithelium, triggering the development of sclerosing cholangitis [1]. Prone positioning of the patient in the ICU might have an additive effect [2]. In reported cases of SC-CIP in literature, the number of liver function tests indicating cholestasis increased during the first two weeks after the beginning of mechanical ventilation, which is typical for SC-CIP [2]. If SC-CIP develops, prognosis appears to be poor, as SC-CIP is associated with high mortality in the early disease course, owing to multi-organ dysfunction syndrome (MODS), and, often liver transplantation will be required if the patient survives the initial life-threatening disease [3]. Until now, there have been reports of 78 patients developing SC-CIP in the literature, mainly as case reports or case series. In these reports, however, a great variety of underlying causes of life-threatening disease requiring prolonged intensive care treatment were described, hindering a risk-factor analysis for the development of SC-CIP. All patients reported herein suffered from life-threatening acute respiratory distress syndrome (ARDS) due to influenza A (H1N1) pneumonia, that is, the same underlying disease. Furthermore, retrospective analysis of these patients revealed not only two groups differing in the development of SC-CIP (approximately 25% with SC-CIP) but also very homogenous patient characteristics in both groups, with no significant differences in pre-existing comorbidities, nor any significant difference in clinical presentation on admission day except for higher body mass index (BMI). Hence, with these excellent prerequisites, we hypothesized that detailed comparison of both groups might uncover risk factors for the development of SC-CIP.

## Methods

This retrospective study was approved by the local ethics committee. Individual informed consent was waived by the local ethics committee for this retrospective study.

### Patients

Twenty-three patients with ARDS and positive polymerase chain reaction for influenza A (H1N1) in the winter seasons 2009/2010 and 2010/2011 were admitted to our ICU. Twenty-one patients (5 female, 16 male, 46.3 ± 10.8 years) were included in this retrospective analysis. Because SC-CIP was shown to develop only after prolonged intensive care treatment, two patients had to be excluded from the retrospective analysis as they had died within a fortnight after admission, owing to intracerebral bleeding and persisting hypoxemia with contraindication to extracorporeal lung support. The remaining 21 patients were divided into two groups as follows: patients who developed SC-CIP (SSC-group) and patients who did not develop SC-CIP (nSSC-group). Diagnosis of SC-CIP was based on four criteria according to Benninger [4]: (i) severe, life-threatening trauma or disease; (ii) slowly increasing signs of cholestasis with a secondary moderate rise of aminotransferases, in contrast with ‘shock liver’; (iii) typical primary sclerosing cholangitis (PSC)-like appearance of the intrahepatic bile ducts with multifocal strictures and dilatations in endoscopic retrograde cholangiography (ERCP); (iv) exclusion of other liver diseases. According to these criteria, five patients (5 male, 0 female, mean age 46.4 ± 9.7) fulfilled all diagnostic criteria of SC-CIP. Sixteen patients (11 male, 5 female, mean age 46.2 ± 11.4) did not develop SC-CIP. Twenty-four days after admission, one patient of the SSC-group died of a cerebral hemorrhage before a typical PSC-like appearance could be confirmed by ERCP; in this patient, however, all other criteria for SC-CIP were fulfilled and histopathologic post-mortem findings confirmed secondary sclerosing cholangitis (SSC).

### Clinical data

Clinical data and laboratory values on the day of admission, days 1, 2, 3, and 7 after admission, and weekly thereafter until day 49, were extracted from comprehensive clinical documentation. Since all patients had been transferred to our tertiary university hospital after initial treatment and intubation in external hospitals, the time of admission to the external hospital and ICU and the time of intubation were also documented. According to the standard operating procedure for patients with ARDS admitted to our ICU, cranial, thoracic, and abdominal computed tomography (CT) data were acquired on the day of admission to the tertiary hospital in all but one patient. Data on age, sex, height, body weight, underlying medical conditions, clinical scores, and clinical outcome were collected. Height was measured manually and body weight was either taken from the protocol of admission of the primary care facility or requested from next of kin. Clinical data included duration of ventilation, respirator settings, blood gas analysis, frequency, and duration of each period of prone positioning, and cumulative time spent in the prone position. The clinical decision for deploying the prone position was based on radiological findings (thoracic CT-scan in the beginning followed by daily X-rays of the thorax), the ratio of arterial oxygen concentration to the fraction of inspired oxygen (PaO_2_/FiO_2_), and increase of PaO_2_/FiO_2_ in the prone position. There was no upper limit for pronation maneuvers. Hemodynamic parameters (arterial and central venous blood pressure, heart rate), catecholamine and fluid therapy, anti-infective therapy, and supportive medication (for example, selenium), as well as the need for venovenous extracorporeal membrane oxygenation (ECMO), duration of ECMO, and required blood flow were recorded.

The severity of illness was assessed using the new Simplified Acute Physiology Score (SAPS II) [5] and Sepsis-related Organ Failure Assessment (SOFA) score [6]. The severity of ARDS was assessed with the Lung Injury Score [7]. Endpoints of this study were discharge from the ICU, day 49, or death.

### Laboratory values

Inflammatory parameters (C-reactive protein, interleukin-6, leukocytes), liver function tests (alanine transaminase (ALT), aspartate transaminase (AST), alkaline phosphatase (AP), total bilirubin (TBIL), γ-glutamyl transpeptidase (GGT)), and oxygen supply (arterial blood oxygen content, blood lactate concentration) were included in the analysis. In addition, blood concentrations of anti-nuclear antibody, perinuclear antineutrophil cytoplasmic antibody, and procollagen peptide III were measured on the day of admission.

### Multi-detector computed tomography (MDCT)

All but one patient underwent (in addition to thoracic scanning) abdominal contrast-enhanced MDCT scanning (64-MDCT SOMATOM Sensation, 128-MDCT SOMATOM Definition AS, Siemens, Erlangen, Germany; 64-MDCT Brilliance, Philips, Eindhoven, The Netherlands) at the time of admission (5 mm slice thickness, 20 or 70 s after intravenous administration of non-ionic iodine contrast media corresponding to the arterial or portal venous phase, 1.5 ml/kg body weight, followed by 100 ml of saline, 3 ml/min flow, (Imeron 350, Bracco SpA, Milan, Italy). All images were reviewed in the soft tissue and bone window.

### MDCT-derived parameters

The following parameters were taken from the abdominal MDCT: the presence of ascites, atherosclerosis, or calcifications at the origin of the intra-abdominal arteries; the diameter of the common hepatic artery; and the presence of intra- or extrahepatic cholestasis. The sagittal diameter of the abdomen was measured at the L4/5 level. In addition, the diameter of the anterior abdominal subcutaneous fatty tissue was measured at the same level, as well as the horizontal intraperitoneal diameter (the distance between both transverse abdominal muscles at the L4/5 level). Furthermore, the volumes of abdominal fatty tissue (subcutaneous, intraperitoneal, and retroperitoneal fat) and of the intra- and retroperitoneal fat only were quantified by manual segmentation, using commercial software (syngo, version VE32B on an MMWP workstation running Somaris 2008G, Siemens HealthCare, Forchheim, Germany). For this evaluation, upper (−30 Hounsfield units) and lower (−300 Hounsfield units) density values were defined to represent fatty tissue. For this semiautomatic approach, slices from the diaphragm down to the caudal border of the pubic symphysis were included. Manual outlining followed the anterior, lateral, and posterior peritoneal walls. The volume of the abdominal subcutaneous fatty tissue was determined by subtracting the volume of the intra- and retroperitoneal fat from the volume of all abdominal fatty tissue. The ratio of the intra- and retroperitoneal fat volume to the overall abdominal fat volume was calculated. To explore whether MDCT-derived thoracic measurements of subcutaneous and intrathoracic fatty tissue give reliable markers for obesity (and could be acquired during thoracic scanning only) and are potential risk factors for developing SC-CIP, the anterior to posterior diameter of the anterior thoracic subcutaneous fatty tissue was measured from the cutis ventral to the manubrium sterni at the level of the first sternocostal joint, and the maximum diameter of fatty tissue from the dorsal to the manubrium of the sternum to the aortic arch (retrosternal fat) was measured.

### Statistical analysis

All data were tested for normal distribution using the Shapiro-Wilk test. Parametric data were reported as mean ± SD, non-parametric data as median with 25th and 75th percentiles. Depending on the data distribution, inter-group comparisons were made using either the student’s *t* test, Fisher’s exact test, or the Wilcoxon rank-sum test. Changes over time were analyzed using the Friedman rank-sum test. In case of significant differences, the Wilcoxon signed-rank test was deployed for *post-hoc* comparison, followed by Bonferroni’s *P* adjustment. A value of *P* < 0.05 was considered significant. Statistical analyses were carried out using the software package R 2.11.1 (The R Project for Statistical Computing).

## Results

### Clinical data and course

In all patients, a diagnosis of H1N1 infection was established by positive H1N1 PCR, either from respiratory or nasal secretions. Of the 21 patients analyzed, five patients (23.8%) developed SC-CIP. Detailed patient characteristics on the admission day are listed in Table 1. In brief, most patients had at least one comorbidity, that is, arterial hypertension in four (19%) and diabetes in three patients (14%). One patient suffered from recently diagnosed but untreated chronic lymphatic leukemia. Five patients (24%) had a history of smoking. No patient had a history of alcohol abuse or history of liver disease. No patient had initial ischemic hepatitis with a high activity of transaminases in the serum or subsequent development of cholestatic parameters. Eight (38%) patients were obese, as defined by the World Health Organization as BMI > 30 kg/m^2^, and ten patients were overweight (48%) (BMI > 25 kg/m^2^). The average BMI was significantly higher in the SSC-group than in the nSSC-group (*P* = 0.03). Average body weight was significantly increased in the SSC-group, as compared with the nSSC-group (*P* = 0.007). Likewise, the average body surface area was significantly increased in the SSC-group, as compared with the nSSC-group (*P* = 0.008). The clinical course of the patients is summarized in Table 2.

**Table 1:** Clinical characteristics on admission day

**Characteristics**	**All patients (n = 21)**	**SSC (n = 5)**	**nSSC (n = 16)**	***P***
Age (years)	46.3 ± 10.8	46.4 ± 9.7	46.2 ± 11.4	NS
Sex				
Male	16	5	11	
Female	5	0	5	
Height (cm)	177.6 ± 8.3	180.0 ± 3.5	176.5 ± 9.5	NS
Weight (kg)	98.4 ± 22.7	120.8 ± 18.1	91.4 ± 20.1	<0.01
BMI (kg/m^2^)	31.2 ± 7.4	37.4 ± 6.0	29.6 ± 7.0	0.03
BSA (m^2^)	2.2 ± 0.3	2.5 ± 0.2	2.1 ± 0.2	<0.01
SAPS II^a^	40.4 ± 9.2	43.4 ± 10.2	39.7 ± 9.3	NS
SOFA*	12.8 ± 1.9	14.0 ± 0	12.4 ± 2.2	NS
Murray	3.5 ± 0.2	3.6 ± 0.3	3.5 ± 0.2	NS
PaO_2_/FiO_2_	90.0 ± 37.9	93.9 ± 44.5	90.6 ± 37.7	NS
PEEP (cmH_2_O)	16.3 ± 2.4	17.6 ± 2.9	16.0 ± 2.2	NS
Peak inspiratory pressure (cmH_2_O)	30.0 ± 4.3	32.0 ± 6.5	29.1 ± 3.4	NS
Lung compliance (l/cmH_2_O)	33.8 ± 11.5	40.2 ± 19.8	31.2 ± 7.2	NS
Ventilation before admission (days)	2.8 ± 3.2	2.8 ± 3.3	3.0 ± 3.3	NS

^a^The Glasgow Coma Scale (GCS) score was not included, since all patients had been intubated and sedated on admission; data for GCS before intubation were not available. BMI, body mass index; BSA, body surface area; NS, non-significant; nSSC, no secondary sclerosing cholangitis; PaO_2_/FiO_2_, partial arterial oxygen pressure/inspired oxygen concentration ratio; SAPS II, New Simplified Acute Physiology Score; SSC, secondary sclerosing cholangitis; SOFA, sequential organ failure assessment.

**Table 2:** Clinical course

**Characteristics**	**All patients (n = 21)**	**SSC (n = 5)**	**nSSC (n = 16)**	***P***
Total duration of ventilation (days)	30.1 ± 48.4	88.2 ± 76.4	11.9 ± 9.2	<0.01
Total days in ICU	39.6 ± 46.1	89.2 ± 75.5	30.1 ± 15.2	<0.01
ECMO				
Patients treated (%)	10 (47.6%)	3 (60%)	7 (43.8%)	NS
Duration of ECMO (days)	27.3 ± 20	45.7 ± 26	19.4 ± 11.5	0.05
Survival (%)	15 (71.4%)	0 (0%)	15 (93.8%)	<0.01
Patients requiring RRT during ICU treatment from day 1 to 28 (%)	10 (47.6%)	4 (80%)	6 (37.5%)	NS
Patients receiving stress-dose steroids (%)	14 (66.7%)	5 (100%)	9 (56.3%)	<0.01
Mean dosage of norepinephrine day 1 to 7 (μg(kg min))	0.13 ± 0.12	0.15 ± 0.12	0.12 ± 0.11	NS

ECMO, extracorporeal membrane oxygenation; NS, non-significant; nSSC, no secondary sclerosing cholangitis; RRT, renal replacement therapy; SSC, secondary sclerosing cholangitis.

### Respiratory function

**Figure 1:**
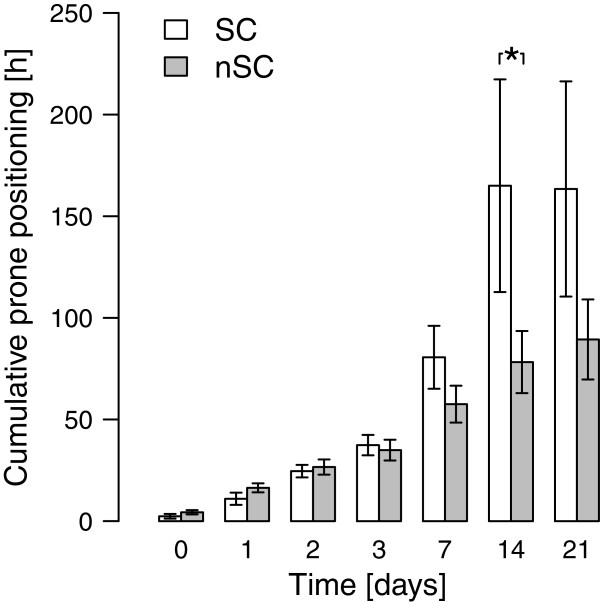
**Cumulative time of pronation in patients without (nSSC) and with (SSC) secondary cholangitis.** * indicates significant difference (*P* = 0.04).

**Figure 2:**
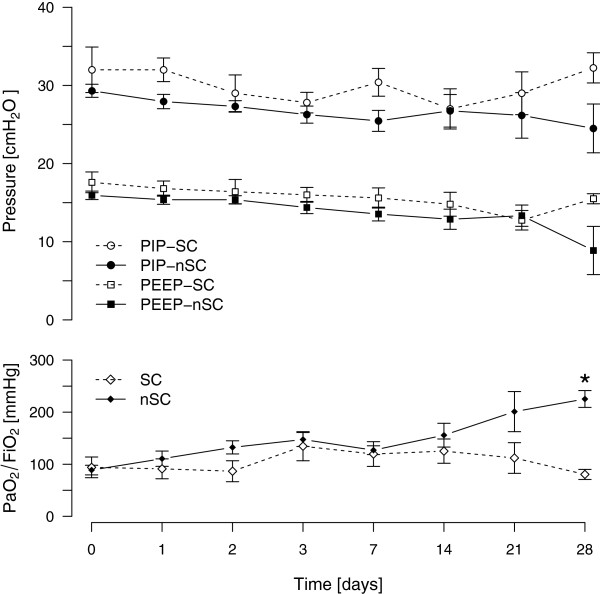
**Ventilator settings and respiratory function indicated by peak inspiratory pressure (PIP), positive end-expiratory pressure (PEEP) and ratio of partial arterial oxygen pressure to the fraction of inspired oxygen (PaO_2_/FiO_2_).** * indicates significant difference (*P* < 0.001).

All patients in both groups required mechanical ventilation, owing to severe pulmonary impairment. Protective ventilator strategy with low tidal volume ventilation (≤6 ml/kg body weight) and application of PEEP levels according to the recommendations of the ARDS Network Group was used. There was no difference in ventilator settings (PEEP/peak inspiratory pressure) or development of dynamic compliance in pressure-controlled ventilation during the first 7 days. Ten patients (48%) required ECMO therapy. In all but one patient, prone positioning was performed to improve oxygenation. The mean duration of a single pronation maneuver was 12 hours in both groups. The SSC-group spent more cumulative hours in the prone position from admission to day 14 than did the nSSC-group (*P* = 0.038) (Figure 1). Oxygenation improved very slowly in both groups (Figure 2).

### Circulatory function

Catecholamine therapy was necessary in all patients on the day of admission and on the following three days. There was no difference in dosage until day 14. The need for more than one catecholamine or vasopressor (that is, norepinephrine and epinephrine ± dobutamine ± vasopressin) was comparable in both groups. Lactate in arterial blood analysis was slightly elevated above the reference range in both groups during the first week but did not differ significantly. The number of patients receiving renal replacement therapy (RRT) and the frequency of RRT were equal in both groups during the first week. After this time, the SSC-group needed RRT more frequently, reaching statistical significance only after day 28. The blood oxygen content was equal in both groups. The shock index did not differ at any time. The mean arterial pressure was significantly higher in the SSC-group on admission day than in the nSSC-group (*P* = 0.041); however, there was no significant difference thereafter.

### Laboratory values

**Figure 3:**
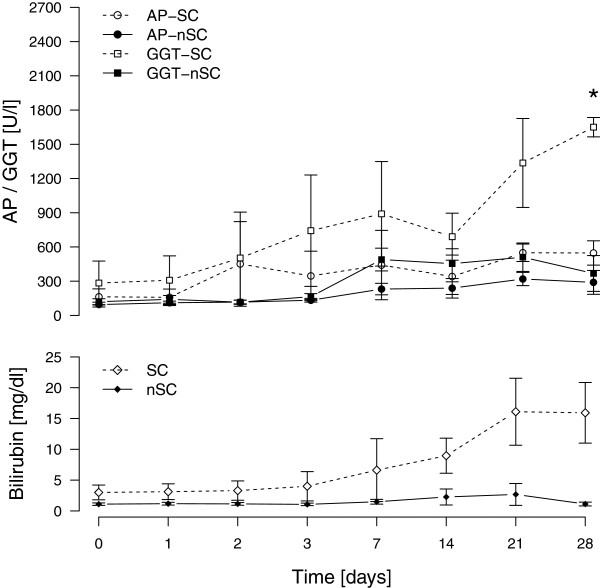
**Temporal course of alkaline phosphatase (AP), γ-glutamyl transpeptidase (GGT) and total serum bilirubin levels in patients without (nSSC) and with (SSC) secondary cholangitis.** * indicates significant difference (*P* = 0.001).

No patient had a history of liver disease. Screening for active viral hepatitis (A, B, and C) produced negative results in all patients. The cholestasis parameters of GGT, AP, and TBIL concentration increased within the first two weeks in the typical pattern of SC-CIP in the SSC-group. After Bonferroni correction, GGT concentration was found to be significantly higher on day 28 in the SSC-group than in the nSSC-group (1650 ± 147 U/l vs. 368 ± 351 U/l; *P* = 0.001). Levels of AP and TBIL were not significantly different after Bonferroni correction (Figure 3) [4,8-10]. Activity of the enzymes ALT and AST in serum were comparable in both groups. Levels of anti-nuclear antibody and perinuclear antineutrophil cytoplasmic antibody were negative in all patients [11]. Plasma procollagen peptide III concentration, a marker of collagen synthesis, measured on admission showed no relevant elevation in either group [12].

The inflammatory parameters C-reactive protein, interleukin-6, and leukocytes were elevated in both groups on admission day without any group difference.

There was no group difference in antibiotic and antiviral treatment during the first two weeks.

### Computed tomography

The results of the evaluation of the CT data are presented in Table 3. In brief, the abdominal fat distribution, as measured by sagittal and horizontal abdominal diameter at the L4/5 level was characterized by higher diameters in the SSC-group than in the nSSC-group (*P* = 0.015 and *P* = 0.003, respectively). Cumulative abdominal fat volumes did not reach significant difference, but there was a difference in volumes of intraperitoneal fat, with a higher volume in the SSC-group than in the nSSC-group (*P* = 0.03). There was no group difference in any of the parameters of thoracic fat measurement. Intra- or extrahepatic cholestasis was not seen in either group. Sludge or concrements in the gallbladder were present in two patients of the nSSC-group. No significant group difference emerged from the measurements of the diameter of the common bile duct, the size of the liver, or the size of the spleen.

**Table 3:** Parameters derived from multi-detector computed tomography analysis

**Characteristics**	**All patients (n = 20)**	**SSC (n = 5)**	**nSSC (n = 15)**	***P***
Abdominal diameter, sagittal (cm)	28.0 ± 4.7	32.3 ± 4.3	26.6 ± 4.0	0.015
Intraperitoneal diameter, horizontal (cm)	28.3 ± 3.7	32.3 ± 4.1	27.0 ± 2.5	0.003
Cumulative abdominal fat (cm^3^)	17317 ± 8683	23677 ± 13759	15197 ± 5382	0.056
Intraperitoneal fat (cm^3^)	5916 ± 2926	8273 ± 3659	5131 ± 2268	0.033
Intraperitoneal fat / height (cm^3^/cm)	33.3 ± 16.1	46.0 ± 20.3	29.0 ± 12.4	0.036
Subcutaneous fat (cm^3^)	11401 ± 6284	15404 ± 10261	10067 ± 3986	NS
Diameter of common hepatic artery (cm)	0.65 ± 0.1	0.65 ± 0.11	0.65 ± 0.1	NS
Diameter of portal vein (cm)	1.4 ± 0.2	1.5 ± 0.3	1.4 ± 0.1	NS
Diameter of inferior vena cava (cm)	2.7 ± 0.3	2.9 ± 0.4	2.7 ± 0.3	NS
Arthrosclerosis (n)	7 (37%)	2 (40%)	5 (34%)	NS
Calcification of celiac trunk (n)	3 (16%)	0 (0%)	3 (20%)	NS
Ascites (n)	9 (47%)	2 (40%)	7 (47%)	NS

NS, non-significant; nSSC, no secondary sclerosing cholangitis; SSC, secondary sclerosing cholangitis.

## Discussion

The homogenous cohort of patients with H1N1-associated ARDS, reported herein, provided a unique opportunity to search for risk factors for the development of SC-CIP, a disease of probably underestimated prevalence and often fatal consequence. The analysis revealed abdominal obesity and increased intraperitoneal fat volume, as well as a longer cumulative duration of prone positioning, in patients with H1N1-associated ARDS who developed SC-CIP, compared with those who did not develop SC-CIP, suggesting these parameters to represent risk factors for the development of SC-CIP. Until now, only case reports or small case series of SC-CIP have been published [2-4,8-10,13-16]. So far, there has been no possibility of comparing the SC-CIP patients with a control group, since the published cases were characterized by inhomogeneous underlying causes for critical illness and were collected over a long period of time (Table 4). Compared with published data of the clinical course of patients with H1N1-associated ARDS; however, our patient cohort showed a similar severity of disease, course of treatment, and outcome [17-21]. The homogeneity of our population allowed for a group comparison between patients with H1N1-associated ARDS who developed SC-CIP and those who did not develop SC-CIP. The laboratory and ERCP findings confirmed the diagnosis for the only recently described entity of SC-CIP in approximately 25% of the whole patient cohort reported herein [1,4]. Obesity was shown to lead to more frequent hospital admissions and to be associated with more comorbidities, but no increase in ICU mortality was reported in medical patients in a meta-analysis of 14 studies with 62,405 critically ill subjects, of whom one-quarter were obese and whose most common causes of admission were respiratory disorders [22]. Morbidity during ICU stay, however, was shown to be increased in obese patients with a significantly higher occurrence of nosocomial infections, a longer period on mechanical ventilation and a longer ICU stay [23,24]. Particularly with regard to hepatic dysfunction, no difference was found in obese patients requiring ICU treatment compared with overweight or normal weight patients [23,24].

**Table 4:** Primary or underlying diseases in previously described critically ill patients with sclerosing cholangitis

**Primary or underlying disease**	***n***
Severe trauma [4,8-10,16]	16
Burns [4,8,10]	11
Extrahepatic bacterial infection [14]	3
Major non-cardiac surgery [8-10]	8
Cardiac surgery [8,10]	10
Power current injury [4]	1
Intracerebral hemorrhage [9]	3
Critical illness without specification [2]	26

The most striking common pre-existing factor of our patient group was obesity or being overweight, which was previously reported to be a risk factor for developing ARDS in H1N1 infection [18,25]. In another study, development of ARDS was seen more frequently in obese patients [26]. In our study, patients with SC-CIP had a significantly higher average body weight and a higher degree of obesity according to their BMI than the patients without SC-CIP. Interestingly, abdominal obesity (measured by the sagittal abdominal diameter with a caliper), rather than a high BMI, was reported to be associated with a higher rate of abdominal compartment syndromes and increased mortality in patients with abdominal obesity requiring ICU treatment [27]. This supports our finding, as there were significantly more cases of extensive abdominal obesity in the SSC-group than in the nSSC-group. Of note, the intraperitoneal fat volume but not the subcutaneous abdominal fat volume was significantly increased in the SSC-group, implying the importance of the intra-abdominal fat distribution, that is, the intra- versus extraperitoneal amount of fat.

In addition, intra-abdominal pressure (IAP), as measured by BMI or sagittal abdominal diameter (SAD), was found to be elevated in critically ill obese patients [28]. Ventilation with PEEP > 15 cm H_2_O, on the other hand, was shown to lead to a mild increase of IAP [29]. Moreover, in the first review of SC-CIP, the authors suspected mechanical ventilation with high PEEP and low tidal volume in combination with vasoconstrictor therapy to promote arterial hypoperfusion of the peri-biliary vascular plexus, causing ischemic injury to the bile duct epithelium, and, hence, triggering the development of sclerosing cholangitis [1].

It was suggested that lying in the prone position would have an additive effect [2]. Interestingly, a mild increase in IAP was described in ventilated patients with acute lung injury lying in the prone position on air-cushioned beds [30]. In that study, there was an increase in cardiac index and mean arterial pressure in patients lying in the prone position with an unchanged plasma disappearance rate for indocyanine green and gastric intramucosal to arterial PCO_2_ in the prone compared with the supine position. Unstable cardiovascular function, however, was an exclusion criterion in that study and, therefore, the effect of the prone position on hepatosplanchnic (dys)function in hemodynamically unstable patients cannot be ruled out. Furthermore, comparison with our results is limited, as the average BMI of the patients in that study (26 ± 3.7 kg/m^2^) was lower than in our SSC-group (37.4 ± 6 kg/m^2^) (*P =* 0.0002). Pronation in ARDS has been discussed in the literature for many years, as a method to improve outcome and has recently been recommended for patients with severe ARDS (PaO_2_/FiO_2_ < 100) [31,32]. In those patients, use of the prone position was suggested, especially if the respective experience in the treatment center was good [33]. There is uncertainty, however, over how long the prone position should be adopted. Mancebo *et al*. [34] described a decreased mortality in patients lying in the prone position over a longer period of time (17 h/d over a mean of 10 d), but Taccone *et al*. [35] could not confirm these results (18 h/session over a mean of 8.4 sessions). In a small series of H1N1 patients, improvement of hypoxemia and limitation of end-organ dysfunction was achieved by pronation in combination with airway pressure release ventilation [17]. All but one patient in our patient groups was placed in a prone position, and this pronation lasted for 12h per session in both groups. Since the cumulative time in the prone position was significantly higher in the SSC-group than in the nSSC-group, this might be a factor increasing the risk of developing SC-CIP. Further studies are warranted to investigate whether abdominally obese patients with ARDS benefit from reduced cumulative duration in the prone position or whether the prone position should be abandoned in these patients.

Michelet *et al*. [36] described a significant increase in IAP and decrease of plasma disappearance rate for indocyanine green if ARDS patients were placed in a prone position on a conventional foam mattress; this was not seen if an air-cushioned mattress was used. In our center, all patients requiring pronation were placed on air-cushioned mattresses. The comparison of the results of our study with those of the study of Michelet *et al*., however, remains limited as their patients showed a lower average BMI of 28.9 ± 5.33, and, hence, their patients were considerably less overweight than the patients of the SSC-group in our population. Another study of patients requiring mechanical ventilation and prone positioning showed an increase of intragastric pressure after 60 min of pronation followed by a significant increase of gastric intramucosal to arterial PCO_2_, indicating insufficient splanchnic perfusion [37]. The body weight or BMI of the patients was not reported in that study. The influence of obesity on IAP in prone position was discussed in a study of Matejovic *et al*., who did not see an increase of IAP or a decrease of hepatosplanchnic blood flow in patients in the prone position [38]. Again the population in this study was less overweight, with a significantly lower BSA than the SSC-group (*P* = 0.00007). To our knowledge, there have been no reports so far of investigation of hepatic function and splanchnic perfusion in obese patients with ARDS lying in the prone position. The higher incidence of need for RRT in the SSC-group after day 28 might be an epiphenomenon as part of the multi-organ failure maintained by unresolved critical illness due to progressive liver failure.

Recently, a compromise of hepatosplanchnic perfusion has been discussed to promote SC-CIP [1]. In our analysis, however, vasopressor therapy and arterial lactate were similar in the SSC- and nSSC-groups. Recently, the administration of norepinephrine in pigs to achieve a mean arterial pressure of 75 mmHg compared with 60 mmHg in the control group did not reduce hepatic arterial blood flow, hepatic venous oxygen saturation, or mesenterial lactate concentration [39]. Furthermore, increasing norepinephrine to achieve a higher mean arterial pressure in vasodilatory shock after cardiac surgery or sepsis did not reduce intestinal mucosal perfusion, tissue oxygenation, or microvascular flow [40,41]. In a study on mechanically ventilated adults, higher levels of PEEP (10 to 20 cm H_2_O), in combination with adrenergic support because of ARDS and septic shock, did not compromise gastric mucosal perfusion [42]. Hence, taking those and our results (no group difference in arterial lactate nor vasopressor therapy) together, non-specific parameters for organ perfusion, such as lactate in arterial blood, shock index, or mean arterial pressure, apparently do not allow a conclusion on hepatic arterial perfusion and, hence, might not be taken into account in the decision whether or not repetitive prolonged pronation in obese patients should be undertaken. Considering the results of our study, a more cautious approach to prone positioning of morbidly obese patients should be considered. Therefore, extracorporeal lung assistance might be an alternative therapeutic strategy in severe ARDS in obese patients. There are no specific studies evaluating the use of extracorporeal lung assist in this patient population.

Wagner *et al*. looked for pre-procedure risk factors to predict the outcome in pulmonary ECMO. They found a trend towards a poorer outcome in patients with a higher BMI [43]. With obesity being epidemic in many developed countries, we are in urgent need for studies clarifying the question of whether morbidly obese patients with ARDS profit from interventional lung assist. The main limitation of our study is the small sample size. Nevertheless, given the rarity of SC-CIP and the high incidence of SC-CIP in our population, we feel that the correlation of obesity and prone position with SC-CIP is worth reporting.

## Conclusions

Considering our findings, obesity, and, in particular, the amount of intraperitoneal fat, and a long cumulative duration spent in the prone position appear to be risk factors for developing SC-CIP. As body weight, intraperitoneal fat, and abdominal fat distribution are factors that elude direct or rapid therapeutic intervention, the positioning of the patient in the ICU remains the decisive factor to focus on. This leads us to recommend monitoring the laboratory parameters GGT and AP on a daily basis in patients with ARDS in the ICU who need pronation, and to re-position the patients should these parameters increase.

## Abbreviations

ALT: Alanine transaminase; AP: Alkaline phosphatase; ARDS: Acute respiratory distress syndrome; AST: Aspartate transaminase; BMI: Body mass index; BSA: Body surface area; CT: Computed tomography; ECMO: Extracorporeal membrane oxygenation; ERCP: Endoscopic retrograde cholangiography; GCS: Glasgow Coma Scale; GGT: γ-glutamyl transpeptidase; IAP: Intra-abdominal pressure; MDCT: Multi-detector computed tomography; MODS: Multi-organ dysfunction syndrome; NS: Non-significant; nSSC: No secondary sclerosing cholangitis; PaO_2_/FiO_2_: Ratio of arterial oxygen concentration to the fraction of inspired oxygen; PCR: Polymerase chain reaction; PEEP: Positive end-expiratory pressure; PSC: Primary sclerosing cholangitis; RRT: Renal replacement therapy; SAD: Sagittal abdominal diameter; SAPS II: New Simplified Acute Physiology Score; SC-CIP: Sclerosing cholangitis in critically ill patients; SOFA: Sepsis-related organ failure assessment score; SSC: Secondary sclerosing cholangitis; TBIL: Total bilirubin.

## Competing interests

The authors declare that they have no competing interests.

## Authors’ contributions

TW designed the study, collected, assembled analyzed and interpreted the data and drafted the article. MIS designed the evaluation of CT data, collected, evaluated, and analyzed the CT data and drafted the article. MED analyzed, interpreted and revised the data and the manuscript. LF, TJ, JM, NG, and MI revised the manuscript, contributing important intellectual content. All authors read and approved the final manuscript.
